# Three steps towards comparability and standardization among molecular methods for characterizing insect communities

**DOI:** 10.1098/rstb.2023.0118

**Published:** 2024-06-24

**Authors:** Ela Iwaszkiewicz-Eggebrecht, Vera Zizka, Christina Lynggaard

**Affiliations:** ^1^ Bioinformatics and Genetics Department, Swedish Museum of Natural History, PO Box 50007, Stockholm, 104 05, Sweden; ^2^ Leibniz Institute for the Analysis of Biodiversity Change, Museum Koenig Bonn, 53113, Germany; ^3^ Section for Molecular Ecology & Evolution, Globe Institute, Faculty of Health and Medical Sciences, University of Copenhagen, 1353 Copenhagen, Denmark

**Keywords:** comparability, metadata, COI synthetic spike-in, metabarcoding, internal standard, multiplex

## Abstract

Molecular methods are currently some of the best-suited technologies for implementation in insect monitoring. However, the field is developing rapidly and lacks agreement on methodology or community standards. To apply DNA-based methods in large-scale monitoring, and to gain insight across commensurate data, we need easy-to-implement standards that improve data comparability. Here, we provide three recommendations for how to improve and harmonize efforts in biodiversity assessment and monitoring via metabarcoding: (i) we should adopt the use of synthetic spike-ins, which will act as positive controls and internal standards; (ii) we should consider using several markers through a multiplex polymerase chain reaction (PCR) approach; and (iii) we should commit to the publication and transparency of all protocol-associated metadata in a standardized fashion. For (i), we provide a ready-to-use recipe for synthetic cytochrome *c* oxidase spike-ins, which enable between-sample comparisons. For (ii), we propose two gene regions for the implementation of multiplex PCR approaches, thereby achieving a more comprehensive community description. For (iii), we offer guidelines for transparent and unified reporting of field, wet-laboratory and dry-laboratory procedures, as a key to making comparisons between studies. Together, we feel that these three advances will result in joint quality and calibration standards rather than the current laboratory-specific proof of concepts.

This article is part of the theme issue ‘Towards a toolkit for global insect biodiversity monitoring’.

## 1. Introduction

Insects represent one of the most diverse and ecologically significant groups of animals on Earth [1]. Accurate and efficient identification of insect species underpins numerous scientific disciplines, including ecology, evolutionary biology, agriculture and medical entomology [2–4]. While traditional morphological techniques have historically dominated insect taxonomic studies, these methods are limited by the availability of skilled taxonomists, the existence of cryptic species, and the degradation of morphological features in fragmented or preserved specimens [5]. DNA-based molecular methods, such as DNA barcoding and metabarcoding, play a crucial role in expediting taxonomic identification owing to their easy implementation and high discriminatory power [6,7]. While DNA barcoding is primarily employed for individual species identification [8], it has also recently been adapted for high-throughput specimen-based barcoding [9]. However, currently, DNA metabarcoding stands as the predominant method used for community-level characterization [10]. In metabarcoding, DNA is isolated directly from mixtures of different specimens and species (bulk samples) or the samples' fixative [11]. DNA traces shed in the environment by organisms can also be extracted from environmental substrates (e.g. soil, water, air), yielding so-called environmental (e)DNA metabarcoding [12]. Subsequently, taxonomically informative DNA regions—barcodes—are mass-amplified and sequenced in parallel using high-throughput technologies (HTS). By bioinformatical analysis of sequences and by comparisons to reference databases, we may thus identify a broad spectrum of species present in those complex samples.

The field of metabarcoding is rapidly and continuously developing, but there is little agreement on basic methodological choices and standard operating procedures. In this context, the standardization and harmonization of technical protocols and data reporting is a pressing issue [13,14]. Only by such harmonization may we ensure the reproducibility of research, large-scale data synthesis and the transfer into applied biodiversity monitoring [15,16]. Recent work by Arribas *et al*. [17] and Chua *et al*. [18] highlighted the fact that current metabarcoding procedures vary tremendously from one study to another. In response, these authors called for efforts to develop standard procedures. Nonetheless, achieving protocol standardization is challenging owing to the fast-evolving and diverse nature of the field. Since current approaches are marked by high variability of techniques and sample types, it would require a great effort to arrive at a universally agreed method for the metabarcoding of insect diversity. Neither may such streamlining be wholly desirable, as the constant development of new approaches is a key motor for driving the field forwards.

Nonetheless, the current state of the art is also an obstacle to the advancement of global insight into the insect fauna through DNA metabarcoding. Most importantly, it hampers comprehensive analyses across studies. Since different methods come with different biases and different resolution, we lack a common benchmark for gauging study-specific patterns against each other. For other fields of analytical science, the current state of metabarcoding would be inconceivable. Consider analytical chemistry. Here, all calibration is based on comparison to a set of samples of known contents—and in support of one's findings, one would naturally report on the precision of one's measures. In metabarcoding, such calibration is typically made against a study-specific sample (or ‘mock community’; [19–21]) of a composition purpose-invented by the laboratory in question. In the end, we face a high probability of comparing apples to oranges.

**Figure 1.**
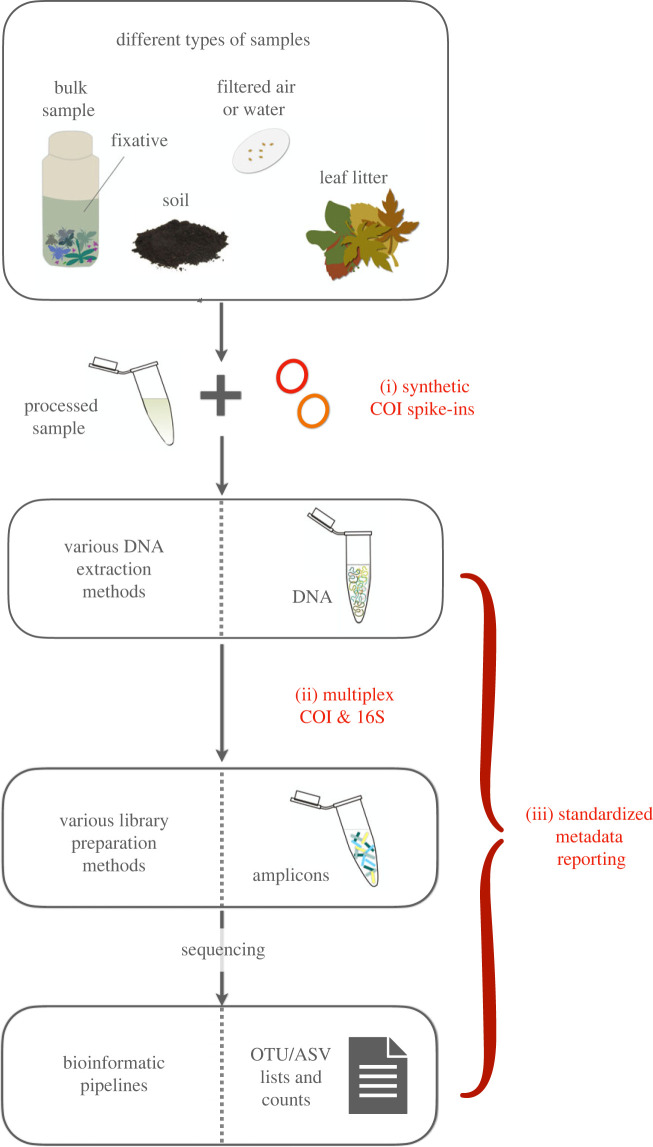
Schematic metabarcoding workflow with the proposed attempts of harmonization highlighted in red. While the overall procedure is variable (different substrates, DNA extraction and libraries preparation methods, bioinformatic pipelines), three proposed approaches for improved standardization can be adopted: (i) integration of artificial spike-ins, (ii) consistent marker gene amplification during PCR and consideration of multiplex approaches, and (iii) standardized reporting of metadata. OTU, operational taxonomic unit; ASV, amplicon sequence variant.

We are then confronted with a situation where biodiversity measurements are urgently needed and new methods are constantly being developed, but joint standards seem out of reach. As an alternative to rigorous standardization which implements the usage of strictly frameworked and compulsory protocols, we feel that the preferred route may be another one: that we should aim for at least a minimum set of recommendations which may in themselves promote data comparability. Therefore, standardization here refers to the integration of quality and reporting standards in metabarcoding protocols and not the streamlining of protocols themselves. In this regard, we discuss: (i) universal internal standards for normalization as well as quality assessment and provide an easy-to-use synthetic spike-in protocol that can be used in metabarcoding assays; (ii) the need for consent on a group-specific marker gene, but also the implementation of multiplex polymerase chain reaction (PCR) approaches, thereby achieving more comprehensive taxonomic coverage; and (iii) the necessity of minimum metadata requirements, and for open-access availability (figure 1).

Overall, these considerations will improve the comparability and reproducibility of insect metabarcoding surveys across samples and experiments, while circumventing the need for strictly enforced standardized guidelines. We are confident that an increased comparability of research will enhance large-scale meta-analysis of data and the integration of molecular approaches in applied surveys.

## 2. Universal internal standards

**Figure 2.**
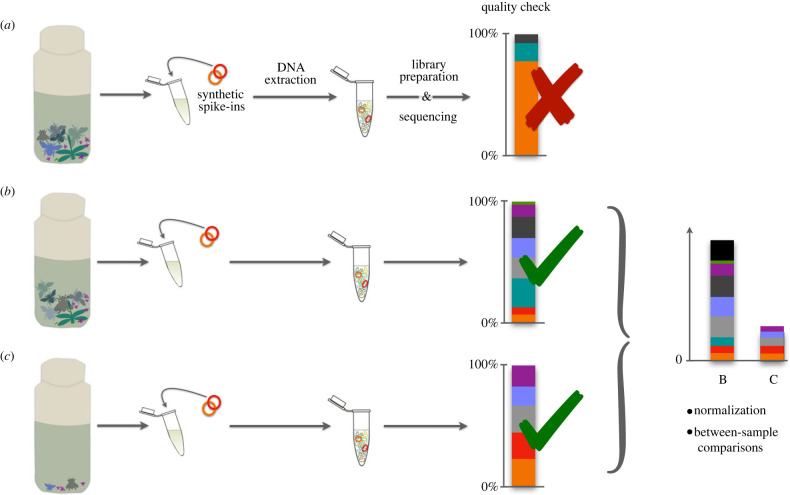
Spike-ins improve quality checks and facilitate normalization. Synthetic spike-ins aid in quality assessment, with deviations in the number and relative proportions of spikes indicating procedural issues (*a*). When spike-ins yield expected proportions of reads (*b*) and (*c*), they can be used for normalization, correcting for amplification and dilutions, and between-sample comparisons.

To arrive at a benchmark for calibration, an increasing number of sequencing studies (i.e. genomics, transcriptomics) rely on the use of so-called ‘spike-ins’ [22–24], also known as internal standards (ISD, [25]). In the context of metabarcoding, this term refers to the deliberate addition of a precise quantity of a known DNA sequence to each experimental sample. The integration of spike-ins into the metabarcoding workflow serves a dual purpose (figure 2). Firstly, they act as sample-specific positive controls, enhancing the evaluation of data quality. Moreover, spike-ins play a pivotal role in decreasing variation that occurs during molecular processing and sequencing. By providing a consistent reference point against which the abundance of a sample's DNA sequences can be gauged, they effectively correct for biases or technical variations introduced during the experimental workflow [25,26]. Bringing all samples to an approximately uniform scale allows, in principle, for meaningful comparisons between samples and experiments. Additionally, this implementation of standards holds the potential to transition from assessing relative abundances to quantifying absolute numbers, thereby overcoming the major limitation of metabarcoding datasets [26–31].

Spike-ins have been successfully applied and proved useful in multiple microbial community studies (reviewed in [25]). For instance, Lin *et al*. [32] spiked-in seawater samples with genomic DNA from yeast and thermophilic bacterium. The authors concluded that spike-ins allowed them to quantitatively compare microbial communities across different samples, as well as to accurately establish phytoplankton chloroplast 16S and genomic 18S rRNA gene abundances. Tkacz and collaborators [30] developed synthetic spike-ins for 16S, 18S and ITS markers, and added them to environmental soil samples. They demonstrated an improved absolute quantification of the microbial community. Despite the utility of spike-ins, they have not gained much use in animal metabarcoding studies, where the dominating marker is a fragment of the cytochrome *c* oxidase gene (COI). So far, only a relatively small number of studies have used COI spike-ins. For instance, Ji *et al*. [27] used a specified mixture of COI barcode amplicon DNA of three insect species to spike-in mock communities as well as standardized environmental samples. They showed that spike-ins performed well as internal standards and improved correction for stochasticity introduced during molecular processing and sequencing. Luo *et al*. [26] used the same three spike-ins as Ji *et al.* [27], adding them to a serially diluted mixture of DNA extracts from various arthropod species (called ‘mock soup’), thus demonstrating that spike-ins helped recovering within-species abundance change between dilutions of the ‘mock soups’. Marquina *et al*. [33] found COI ISD to be a useful tool in assessing the preservation of DNA after long-term storage in a range of ethanol concentrations. They added a spike-in standard right before the DNA amplification step and then compared the ratio of insect amplicon copy numbers to this spike-in.

When considering internal standards for use in insect biodiversity surveys, one should distinguish three types: (i) biological spike-ins—in which insect specimens are added to every sample in the same numbers [21]; (ii) DNA spike-in—in which pre-amplified insect marker DNA is added in a specific amount [26,27]; and (iii) synthetic spike-in—in which artificial DNA molecules, designed *in silico* and synthesized in a laboratory, are added to the sample. For ease of identification, these molecules are designed to lack similarity to any sequence in public databases [24,31].

While each of the spike-in types has its advantages and limitations, there are a few common aspects to consider when searching for universal spike-in for insect studies. Firstly, the principle of internal standard demands that the same amount of the spike-in material is added to each sample. DNA and synthetic spike-ins allow, in principle, for precise measurements of the input material, while biological spike-ins do not permit full control of the amount of input DNA owing to biological differences between spike-in individuals [21]. Secondly, an efficient spike-in must be readily available in almost infinite amounts, both at present and in the future. As we aspire to employ long-term terrestrial insect surveys over decades, we must make sure that the same internal standard will be available in the future. Long-term supply of biological spike-ins is difficult to assure and maintaining populations is costly and time-consuming. DNA and synthetic spike-ins can be amplified into infinity; however, the DNA spike-ins have a limited source (actual insects) and demand careful maintenance. In an event of DNA degradation or material damage, the source can be lost and difficult (or impossible) to recreate. Synthetic spike-ins are free from this problem, as they can be resynthesized anywhere at any moment using commercial services and following standard protocols. Last but not the least, spike-in DNA sequence must be easily distinguished from the sample material being studied. This implies that the species chosen as a source of biological or DNA spike-ins must be carefully selected to ensure they are not present in the region where the study samples come from. This fact precludes the universal, worldwide use of biological or DNA spike-ins. Synthetic spike-ins appear particularly attractive as a solution to this issue, as they are by design different from any biological sequence in the databases and can be added to any sample irrespective of their origin and composition. Taking all this into consideration, and consistent with the conclusions of Harrison *et al*. [25], synthetic spike-ins are the most suitable choice for DNA-based community studies. Nevertheless, to the best of our knowledge, there are currently no universal synthetic spike-ins in use for COI-based metabarcoding biodiversity surveys.

Here, we address this gap by proposing two synthetic COI spike-ins, named *Callio-synth* and *tp53-synth*. *Callio-synth* has already been described and used in the DNA degradation experiment by Marquina *et al*. [33], but for clarity we will summarize the design in brief here. As the starting point for designing both spike-ins, we used a sequence of a bluebottle fly (*Calliphora vomitoria*). We preserved the primer binding sites that match the primers BF3-BR2 [34] and fwhF2-fwhR2n [35] as well as a flanking region around each primer site (±6 bp). In the case of *Callio-synth,* the remainder of the sequence was replaced by a random DNA sequence that does not resemble any known sequence deposited in GenBank, while keeping GC content similar to the original *C. vomitoria* sequence. For the other spike-in*,* we replaced sequences between preserved primer-binding sites with short fragments of the *tp53* gene (*tp53-synth*). Since the *tp53* sequences are very short and used in the context of COI metabarcoding, we do not risk any confusion when analysing results. Both spike-in sequences (471 bp total length) can be easily synthesized by a commercial laboratory anywhere in the world. They should then be inserted into standard plasmids (also commercially available) and transformed into monoclonal bacteria—a stable system for long-term storage and amplification. Spike-ins can be extracted from the bacterial culture whenever needed by commonly used kits. The concentration of spike-in DNA must be quantified and the same number of spike-in copies should be added to every sample before DNA extraction. Subsequently spike-ins are co-extracted, co-amplified and sequenced together with the DNA of the insect sample. A step-by-step protocol providing spike-in sequences and detailed information on design and how to proceed with bacterial cultures and quantifications is available following the link: https://dx.doi.org/10.17504/protocols.io.14egn33ryl5d/v2 (also available as the electronic supplementary material, file S1). More information on how to design a synthetic spike-in is summarized in the review by Harrison *et al*. [25].

As the use of COI synthetic spike-ins is in its nascent stage, many questions naturally remain. As a first question, we may ask whether the use of just two spike-ins is enough? The comprehensive review by Harrison *et al*. [25] suggests that at least three distinct spike-ins should be added to each sample and that evaluating ratios of spike-in reads should become part of the quality control. As a second question, we may consider how much spike-in should be added? The exact amount will depend on the nature of the sample, i.e. whether it has high DNA yields (e.g. homogenized tissue samples) or just trace amounts (i.e. eDNA). The challenge is to have enough reads to make full use of spike-ins while not losing too much sequencing bandwidth on them. The literature review conducted by Harrison *et al*. [25] suggests that one should aim for spike-ins to constitute 1–3% of the total DNA in a sample. Naturally, it is not an easy goal to achieve when processing large numbers of diverse samples. However, dividing samples into distinct size categories, based on insect biomass or the quantity of input DNA, presents a feasible approach for incorporation of suitable levels of spike-ins. In our proposed protocol, developed for a large collection of homogenized Malaise trap samples (originating from the Insect Biome Atlas project, insectbiomeatlas.org), we add approximately five million copies of each spike-in to samples. While this exact procedure can only be recommended for samples of similar type, the rationale and methodology outlined in the protocol can serve as a template or starting point for other studies. Undoubtedly, standards regarding the exact amount of spike-in DNA appropriate for different kinds of samples remain to be developed and agreed upon by the community. A third question relates to how the presence of spike-ins influences community reconstruction of the samples? It is known that presence of one DNA sequence can affect the recovery of other sequences present in the samples [36]. Perhaps the addition of synthetic spike-ins could influence our ability to recover certain COI barcodes? While this aspect remains to be studied, a simple null model is to assume that the same effect will hold across all samples and studies that use them.

The proposed spike-ins are designed to work in experiments targeting two different primer pairs (or combination of those primers). Of these, *BF3-BR2* produces a 418 bp long fragment of the Folmer region and is commonly used in Illumina-based studies, whereas *fwhF2-fwhR2n* allows for the amplification of a shorter fragment, only 205 bp long, which is particularly useful when dealing with degraded DNA. If a different pair of primers is desired, or the study focuses on a full-length barcode, then new synthetic spike-ins will be necessary. The same holds true if a single reaction targets different markers to improve taxonomic resolution (multiplex approaches—see next section). Here synthetic spike-ins for all target fragments should be integrated in the process. We do not claim to have generated the final set of synthetic spike-ins, but merely encourage the community to discuss, test and develop synthetic spike-ins, to thereby bring the whole field into a more rigorously quantitative and standardized future.

We propose that synthetic COI spike-ins should become the standard and be included in all insect surveys, as they are a powerful tool improving the reliability and accuracy of COI metabarcoding. However, we also highlight that the synthetic spike-ins put forward in this paper are merely a starting point. For future development, it will be important to consider expanding the length of the spike-in to cover the whole Folmer region, to design additional spike-ins and to test them in different combinations and concentrations in order to find the optimal standard spike-in mixture for universal use.

## 3. Targeting multiple gene regions

In both DNA barcoding and metabarcoding, it is possible to target and amplify different mitochondrial gene regions (such as COI, and the ribosomal RNA genes 16S, 12S, 18S and 28S) through PCR. Given that these molecular methods rely on databases to assign taxonomy to the sequences obtained, the completeness of the database is crucial for accurate assignment [37]). At present, the database for COI is more complete than that of other regions [38]. Therefore, if using other regions, the taxonomic resolution will be influenced by the database incompleteness. As a further advantage, the COI region presents enough variability between species and sufficiently little variation within species to allow for accurate species-level taxonomic assignment [17]. All this has led researchers to recommend targeting the COI region in insect community metabarcoding (e.g. [39]). Nonetheless, at lower taxonomic resolution, other loci such as 16S rRNA can provide a better overview of the insect community present in the sample. In some cases its use has provided data complementary data to that obtained with COI [40,41]. Because of this, and together with primer bias in the efficiency of PCR amplification [42,43], some researchers recommend and have used more than one primer set [44–47]. Nevertheless, this can be labour intensive and expensive, as it increases the amount of reagents needed for PCR reactions and sequencing if each primer is used in a separate reaction. Therefore, to keep the costs down and to allow comparability between studies, there is consensus on the use of the COI region as the first option [38].

In a multiplex approach, several primer sets are used in the same amplification reaction, which thereby produces multilocus data [6,48]. If the primers are chosen to generate amplicons of different lengths, it is possible to differentiate which sequences belong to which primer [49]. However, multiplexing PCR can present technical challenges especially in mixed samples where the amount of the template DNA differs across taxa. Owing to differences in the signal from these taxa, primer concentration in the reaction mix must be compensated based on their amplification efficiency [50]. In addition, other calibrations should be performed to balance the reaction, such as optimizing thermocycling conditions and making sure there are no cross primer dimers (see [48,51] for more detailed information). The sensitivity and specificity of the assay can be assessed by the use of spike-ins as described in the previous section.

Multiplexing has been successfully used with species-specific primers. For example, it has been used to target and amplify the 16S rRNA and COI region of several species of edible insects [52]. Multiplex has also been implemented using general primers targeting a wider taxonomic spectrum. For example, de Kerdrel and collaborators [53] used multiplex PCR to target four nuclear DNA regions (28SrDNA, V1-2 and V6-7 regions of 18SrDNA and histone H3) in bulk arthropod samples. In evidence of its efficiency, multiplexing has been used to amplify spider DNA through a mitochondrial multiplex assay simultaneously targeting the COI, 12S and 16S regions and a nuclear multiplex assay targeting the D6 region of the 28S gene, the V1-V2 and the V6-V7 regions of the 18S, the ribosomal ITS2 and histone H3 [48]. In addition, the COI, 16S, 18S and 28S genes have been targeted to study the diet of spiders [54], and the COI, 12S and 18S genes for pest insect identification [55]. Batuecas *et al*. [49] used two primers targeting different regions within COI. These studies show that the use of a multiplex approach for arthropod identification is successful, but they also illustrate the lack of consensus about which loci to target.

Although the multiplex method has been applied in several studies, it is still less frequently used than the non-multiplex approach. While Krehenwinkel *et al*. [56] targeted different regions within the COI gene to identify insect DNA in tea bags, we are not aware of other studies implementing multiplex PCR for targeting insect DNA in environmental samples (eDNA). This seems a missed opportunity, as it could accelerate the application of high-throughput surveillance [55], especially if also applied to new sources of insect DNA such as air [47,57], rain water [58] and other novel DNA substrates (reviewed in [18]).

**Figure 3.**
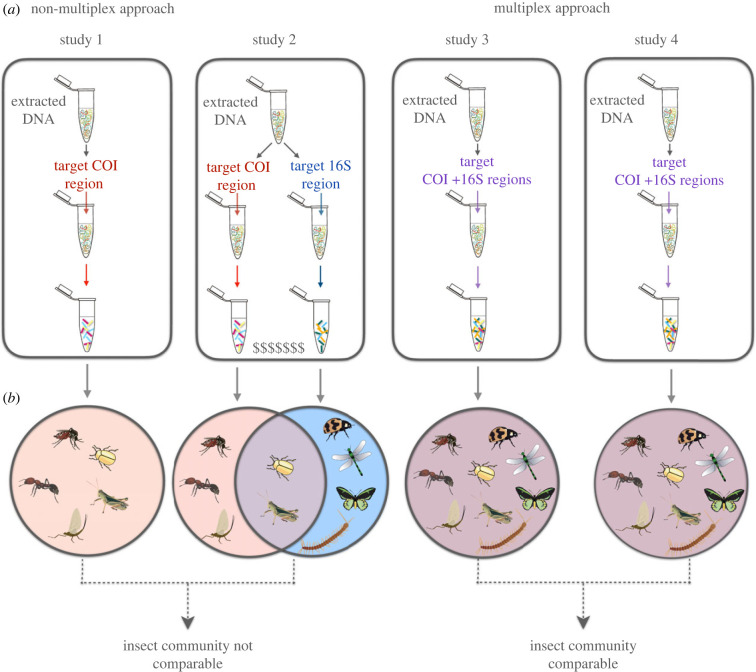
The choice of the gene region targeted in metabarcoding studies will impact the insect community detected and can hinder data comparability across studies. (*a*) Targeting several gene regions using a multiplex approach can be time and cost-effective and (*b*) targeting the same regions can further allow data comparability between different studies. Animal images in the figure obtained from the Integration and Application Network, University of Maryland Center for Environmental Science (ian.umces.edu/symbols/).

Therefore, we suggest that the multiplex approach could be routinely used across different research groups, projects and sample types. The use of several loci can improve the characterization of the community [40] and support phylogenetic analyses [48]. Further, if multiple laboratories were to target the same loci in their multiplex PCR, then this would allow comparability across laboratories (figure 3). In conclusion, we consider that owing to the relative completeness of the reference database and the possibility of obtaining high taxonomic resolution of the sequenced data, it is important to keep targeting the COI gene as a standard solution for metabarcoding. In addition to COI, another primer set targeting a different region should be added in a standard multiplex assay. Beyond COI, the regions most commonly used (also in a multiplex setting) are 18S and 16S. While the 18S region is generally used for a broader taxonomic overview including metazoans [59], the 16S region has been proved to outperform 18S when targeting insect DNA, especially in the detection of dipterans [59]. Further, the 16S region has been found to provide data complementary to that gained from the COI gene [40]. Therefore, we suggest that 16S should be included in the multiplex assay, together with the COI gene. However, we also need to point out that reference databases for this locus should be improved to allow for better taxonomic assignments. Naturally, we encourage the use of other loci, too, but offer COI and 16S as a solid starting point. Finally, we suggest that the use of multiplex PCR will prove useful beyond insects in bulk samples, and should be explored further when targeting small traces of insect DNA in environmental samples.

## 4. Standardization of metadata reports

**Figure 4.**
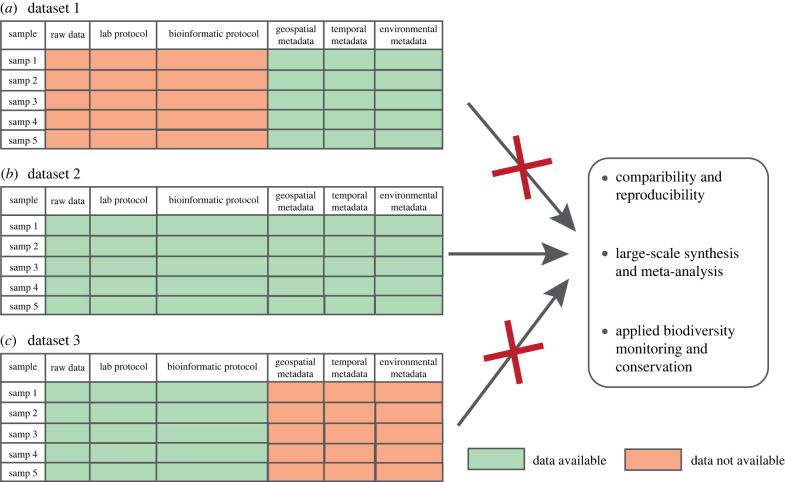
Complete and well reported metadata (*b*) strengthens comparability of different metabarcoding studies and enables meta-analysis. Incomplete and non-published documentation of metadata from metabarcoding community analysis (*a*) and (*c*) prevents comparability of datasets, hampering meta-analysis and large-scale synthesis as well as the integration in applied monitoring schemes and conservation. *Raw data*: link to archive (such as NCBI Sequence Read Archive or EBI-ENA), where raw sequence data are stored also including sample description; *lab protocol*: laboratory protocol information based on the MIMARKS reporting standards (see main text, [72]) and extended in the electronic supplementary material, table S2—laboratory protocol); *bioinformatic protocol:* minimum reporting standards of main programs and parameter settings for bioinformatic sequence analysis as summarized in the electronic supplementary material, table S2—bioinformatic analyses; *geospatial, temporal and environmental metadata:* all sample associated metadata as proposed in MIMARKS standards and associated environments (e.g. through GEOME platform, see main text).

Molecular biodiversity research yields enormous quantities of community-level data, comprising raw sequence material but also associated technical protocols and environmental metadata [17,60]. Accessibility of raw sequences and technical information is a requisite for retrospective data analysis, long-term studies and global syntheses, and so is public access to environmental and ecological metadata [20,61–63]. While centralized publication of raw sequence data is widely adapted in metabarcoding surveys through platforms such as the National Center for Biotechnology Information (NCBI) Sequence Read Archive [64] or the European Bioinformatics Institute Nucleotide Archive (EBI-ENA—also linked with MGnify as a platform for comparative analysis for microbiome data) [62,65,66], the associated metadata are often scarce, inconsistent and/or non-standardized—or remain completely unreported [67–70]. Here, current tools and standards for data deposition remain of little help, as they tend to focus exclusively either on genomic or ecological attributes [62,71]. The unavailability of technical and ecological metadata hampers transparency and reproducibility of research, preventing data exchange and reuse and subsequently the integration of data into large scale meta-analysis (figure 4). This is a major concern, as it restricts access to metabarcoding data by governmental conservation organizations and large-scale monitoring initiatives, which rely on standardized data collection and reporting guidelines [61,68,73,74]. While huge variability in laboratory protocols, bioinformatic pipelines and sample types impedes strict technical standardizations (see Introduction), there is clearly a need for improved reporting standards [17,62,72]. This necessity for streamlined reporting of the metadata behind molecular surveys has been discussed by different consortia and publications (e.g. [17,61,62,67,75]). Nonetheless, given the continuing challenges in making an actual change, we here want to stress the salient points and the key items needed to ensure the true replicability and large-scale synthesis of data.

To remedy the state of the art, several authors have argued for the use of data sheets with at least minimum metadata to be associated with published HTS data [17,62,76] with a focus on eDNA approaches [68,75,77]. However, for large-scale analysis of insects, different amplicon-based methods can be used, and metadata storage should not exclusively focus on eDNA standards. Checklists for minimum information to improve mechanisms of metadata capture and exchange have been initiated by the Genomics Standards Consortium. Those were initially designed for genomic and metagenomic approaches targeting microorganisms, with the attempt to record minimum technology-specific, geospatial and temporal metadata (see [72]). Extant checklists (e.g. ‘minimum information about a marker gene sequence (MIMARKS) (https://genomicsstandardsconsortium.github.io/mixs/, [60,61,72] can be universally applied to any marker gene at levels from single individuals to complex communities and incorporates standards developed by the Consortium for the Barcode of Life (CBOL). Yilmaz *et al*. [72] identified the minimum information to be associated with the sequence data, including geospatial and technical details (e.g. sample collection method and device, sample size, target gene, PCR condition, integrated negative and positive controls). Requirements for minimum information are available and differ slightly for different environmental samples (e.g. water, soil, sediment, air). In brief, rather than reinventing the wheel, we might thus adopt the checklists introduced for minimum sequence information as a first standard for the publication of DNA metabarcoding metadata and reporting. This is also the goal of Genomic Observatories Metadatabases (GEOME, https://geome-db.org), which is an open access platform permanently linking molecular raw data (Sequence Read Archive) with associated sample metadata including technical protocols and environmental information [71].

However, while previous checklists for metadata do include detailed information from sampling to sequencing, the information required about bioinformatic processing and taxonomic assignment of sequences is limited (including steps such as sequence quality check, chimera identification, etc.), especially if no precast pipeline is used ([78] see [79] for detailed review of existing pipelines). Therefore, we here introduce further minimum information about sequence analysis that ensures reproducibility and should be considered for publication in metabarcoding studies in table format (electronic supplementary material, file S2). Notably, such a solution can also reduce the textual portion in the material and methods section. In particular, we solicit data on the programmes and detailed parameter settings used for the different steps of bioinformatic analysis. In addition, the table provided (electronic supplementary material, file S2) includes minimum information about sample replication, integrated positive and negative controls and how those are used in downstream data quality improvement.

While the need for standard open-access metadata publication along with metabarcoding datasets has been repeatedly raised [17,62,69,76], documentation remains poor. By pointing to extant minimum requested checklists and associated platforms, we emphasize the continuing need for open-access publication of environmental and technical data, and for adequate information about bioinformatic analysis. We believe that the availability of geospatial and environmental metadata, and the standardized reporting of the protocols applied, forms the very basis for cross-laboratory meta-analysis of metabarcoding data and the successful transfer of methods into applied monitoring schemes.

## 5. Moving towards comparability and standardization

In the light of the increasing volume of insect DNA data being generated globally, there is undeniable evidence of the successful application of molecular methods in yielding valuable insights. Nevertheless, the question regarding how to create comprehensive insect monitoring schemes still remains, as each study uses different protocols, calibrates the data in different ways and reports the data using different formats. We suggest that rather than pursuing strict standardization of protocols, the adoption of a small number of easy-to-implement standards is crucial. To this end, we propose (i) synthetic spike-ins as internal standards, (ii) emphasis on a standard marker gene but consider additional fragments through multiplex PCR, and (iii) commit to the publication and transparency of all protocol-associated metadata.

To unlock the full potential of molecular methods for characterizing insect communities, global standardization and cross-study comparisons are imperative. We believe that the three suggested advances will help in achieving uniform quality and calibration across different studies, thereby paving the way for large-scale data meta-analysis.

## Data Availability

The protocol detailing use of synthetic spike-ins is published on protocols.io (doi:10.17504/protocols.io.14egn33ryl5d/v2): https://www.protocols.io/view/synthetic-coi-spike-ins-for-use-in-metabarcoding-b-14egn33ryl5d/v2 [80]. Supplementary material is available online [81].
